# Dangerous Alarming Diameter Assessment (DADA Index) in Which the Ratio of Iris Surface/Pupil Surface Size Is More Reliable than Pupil Diameter Measurement in Comatose Patients After Subarachnoid Haemorrhage: An Experimental Rabbit Model

**DOI:** 10.3390/diagnostics15212696

**Published:** 2025-10-24

**Authors:** Hüseyin Findik, Mehmet Dumlu Aydın, Feyzahan Uzun, Muhammet Kaim, Ayhan Kanat, Osman Nuri Keleş, Hakan Hadi Kadıoğlu, Mehmet Emin Akyüz, Mete Zeynal

**Affiliations:** 1 Department of Ophthalmology, School of Medicine, Recep Tayyip Erdogan University, 53100 Rize, Turkey; feyzahan@gmail.com (F.U.); muhammet.kaim@erdogan.edu.tr (M.K.); 2 Department of Neurosurgery, School of Medicine, Ataturk University, 25030 Erzurum, Turkey; nmda11@hotmail.com (M.D.A.); hakanhadi@gmail.com (H.H.K.); mehmeteminakyuz25@gmail.com (M.E.A.); dr.metezeynal@gmail.com (M.Z.); 3 Department of Neurosurgery, School of Medicine, Recep Tayyip Erdogan University, 53100 Rize, Turkey; ayhankanat@yahoo.com; 4 Department of Histology, School of Medicine, Ataturk University, 25030 Erzurum, Turkey; onkeles@hotmail.com

**Keywords:** subarachnoid haemorrhage, DADA index, pupil diameter, Edinger–Westphal nucleus, brain death, neurodegeneration, SD-OCT, rabbit model

## Abstract

**Objective/Background:** Pupil diameter varies across individuals, limiting its reliability in assessing cerebral pathologies, particularly in comatose patients following subarachnoid haemorrhage (SAH). The Dangerous Alarming Diameter Assessment (DADA) index, defined as the ratio of iris surface to pupil surface, may offer a more precise diagnostic tool. This study evaluates the efficacy of the DADA index compared to pupil diameter in predicting neurodegeneration in the Edinger–Westphal nucleus (EWN) and diagnosing brain death in an SAH model. **Methods:** Twenty-two rabbits were divided into Control (*n* = 5), Sham (*n* = 5), and Study (SAH, *n* = 12) groups. Pupil diameter and DADA index values were measured via spectral-domain optical coherence tomography (SD-OCT) in groups at post-intervention (0.75 cc serum physiologic injection for Sham, 0.75 cc autologous blood injection for Study). After one week, animals were sacrificed, and EWN degenerated neuron density was quantified using stereological methods. Data were analysed with Kruskal–Wallis and Mann–Whitney U tests, with correlations assessed for pupil diameter and DADA index against EWN neurodegeneration. **Results:** Pupil diameter assessment classified all 12 study group subjects as deceased, primarily due to fixed and dilated pupils. In contrast, the DADA index identified only 8 of these 12 subjects as deceased, with EWN degenerated neuron density exceeding 80%, while the remaining 4 subjects showed less than 80% neurodegeneration, indicating viability. Strong negative correlations were observed between pupil diameter (r = −0.972, *p* < 0.001) and DADA index (r = −0.977, *p* < 0.001) with EWN neurodegeneration. The DADA index demonstrated superior precision in distinguishing severe neurodegeneration, suggesting its potential as a criterion for brain death assessment. **Conclusions:** The DADA index provides a more accurate and nuanced evaluation of EWN neurodegeneration compared to pupil diameter, offering a promising diagnostic tool for brain death in SAH-induced comatose states, with potential implications for future brain transplantation diagnostics.

## 1. Introduction

The background information for this study highlights that, particularly in emergency and critical care environments, the evaluation of pupillary response and size is still among the most basic and important neurological tests in clinical practice [1,2,3]. Its accessibility and capacity to offer vital information on brainstem function have made pupillary examination a mainstay in neurological evaluation for decades [3,4,5,6]. Though there are several natural constraints and standardisation issues with this method, the conventional test depends mostly on pupil diameter measurements and light responsiveness [3,7,8]. The complex neural route, including both sympathetic and parasympathetic nervous systems, controls the pupillary light reflex; the Edinger–Westphal nucleus (EWN) is fundamental in the parasympathetic regulation of pupil contraction [9]. The EWN, which is situated in the midbrain, is especially sensitive to rising intracranial pressure, compression, and ischaemic damage linked with subarachnoid haemorrhage (SAH) as well as other acute neurological disorders [10,11].

Against this context, the research problem emerges, recognising that although pupillary evaluation is clinically important, current techniques have limitations that compromise diagnostic accuracy. Often neglecting individual anatomical differences in iris and pupil size, standard pupillary assessment mostly emphasises absolute diameter measures [1,2]. Based on age, heredity, and pre-existing eye diseases, among others, these differences can be significant between people [12,13,14]. Moreover, some examiners employing subjective assessment methods under varying illumination circumstances conduct pupillary evaluations in critical care environments [3,15,16]. In comatose patients after subarachnoid haemorrhage, when therapeutic decision-making depends on the pathophysiological relevance of pupillary changes, the interpretation of pupillary changes becomes even more difficult [17,18,19]. This leads to the following central research question: Especially in the context of subarachnoid haemorrhage, could a ratio-based evaluation of pupillary metrics, specifically the link between iris surface area and pupil surface area, provide a more consistent biomarker of neurological damage than traditional pupil diameter measurements alone?

This experimental study’s main goal is to assess and contrast the diagnostic accuracy of the Dangerous Alarming Diameter Assessment (DADA) Index, defined as the ratio of iris surface area to pupil surface area, against conventional pupil diameter measurements in detecting and quantifying neuronal degeneration in the Edinger–Westphal nucleus following subarachnoid haemorrhage. By introducing a novel metric, the study seeks to address the shortcomings of existing methods and explore whether ratio-based measurements can serve as more reliable indicators of underlying neurological injury.

The significance and motivation of the research are underscored by the fact that, while subarachnoid haemorrhage accounts for only about 5% of all strokes, it contributes disproportionately to stroke mortality and morbidity, with case fatality rates exceeding 50% [20,21]. Guiding treatments and enhancing results depend on early and precise neurological examination. Though its present use lacks consistency and accuracy, pupillary evaluation offers a non-invasive window into brainstem activity [5,7]. There is a clinical demand for more consistent and dependable neurological evaluation instruments that may be used at the bedside drives this work. By perhaps removing the confounding consequences of baseline anatomical variances, the creation of ratio-based pupillary measures fills a major void in clinical evaluation [22,23]. Moreover, a more precise pupillary evaluation technique might improve prognostication in important neurological diseases and direct time-sensitive therapeutic decisions on surgical intervention, neuroprotective measures, and end-of-life care [16,24,25].

Expanding on this, the new field contributions of this work are evident as the DADA Index is presented as a new measure with the potential to radically change pupillary evaluation in neurocritical care. This method recognises the natural biological variation in ocular architecture by emphasising the proportional relationships that represent neurological function and by thinking about pupillary alterations as a ratio rather than an absolute value [25,26,27]. Crucially this study also shows a clear link between a clinically visible measure—pupil index—and histological results—neuronal degeneration in the EWN—thereby offering a vital link between bedside evaluation and underlying neuroanatomical damage [16]. The possible use of the DADA Index goes beyond SAH to include other acute neurological diseases and could help to continuously improve brain death criteria [28].

Finally, grounding the study is the theory framework, which is based on a robust neuroanatomical model recognising the multiple pathways involved in pupillary control. Afferent routes via the optic nerve, processing centres in the pretectal nuclei, and efferent parasympathetic pathways via the Edinger–Westphal nucleus and oculomotor nerve comprise the pupillary light response [9]. Subarachnoid haemorrhage can disturb these pathways by several methods, including direct compression, vasospasm, and inflammatory processes [29,30]. The theoretical basis includes both concepts of neuroplasticity and cell death thresholds as pupillary alterations indicate not only the existence of injury but also perhaps the degree and reversibility of neuronal damage. This work intends to create a more strong theoretical foundation for pupillary evaluation in severe neurological diseases by means of a quantification of the correlation between visible pupillary measurements and histological alterations.

## 2. Materials and Methods

This study investigated the effects of experimental subarachnoid haemorrhage (SAH) on pupil dynamics and neuronal integrity in the Edinger–Westphal nucleus of rabbits. A total of 22 rabbits (2.5–3 kg) were utilised. These animals were allocated into three groups: a baseline control group (*n* = 5), a sham group (*n* = 5), and a study group (*n* = 12). Prior to intervention, pupil diameters of all animals were measured using spectral-domain optical coherence tomography (SD-OCT) (Optovue, Inc., Fremont, CA, USA) under direct sunlight. Anaesthesia was induced in the control, sham, and study groups via subcutaneous injection of a cocktail comprising ketamine hydrochloride (25 mg/kg), lidocaine hydrochloride (15 mg/kg), and acepromazine (1 mg/kg). Following preparation of the occipito-cervical region, SAH was experimentally induced in the study group by injecting 0.75 cc of autologous blood, obtained from the auricular arteries, into the cisterna magna. In contrast, the sham group received an intracisternal injection of 0.75 cc of normal saline solution. Animals were monitored for a period of one week, during which pupil diameters were measured daily. Throughout this period, all rabbits were maintained on a standard diet with ad libitum access to water. At the conclusion of the observation period, the animals were humanely decapitated.

### Histological and Statistical Analysis

**Figure 1:**
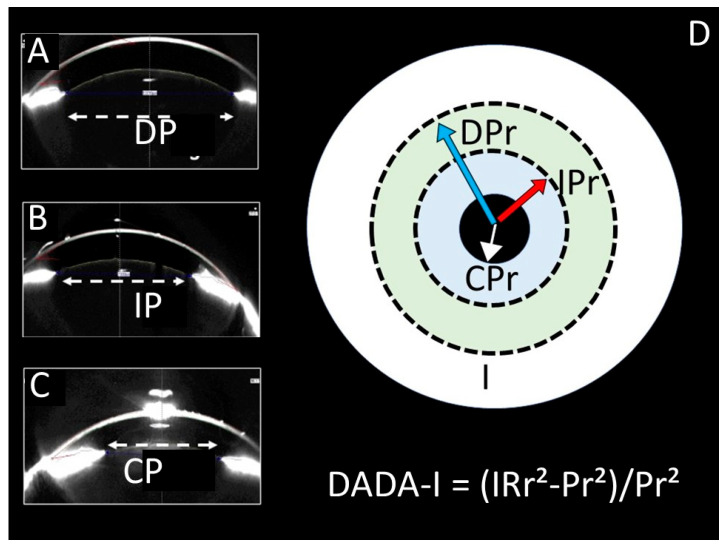
Pupil Measurements and DADA Index Schematic: (**A**) Optical Coherence Tomography (OCT) measurement of a dilated pupil (DP). (**B**) OCT measurement of an isocoric pupil (IP). (**C**) OCT measurement of a contracted pupil (CP). (**D**) Schematic diagram illustrating the components of the DADA Index (DADA-I) calculation, defined as the ratio of the iris surface area (IRr^2^) to the pupil surface area (Pr^2^). The diagram shows the radii for a dilated (DPr), isocoric (IPr), and contracted (CPr) pupil relative to the total iris (I). The formula is: DADA-I = (IRr^2^ − Pr^2^)/Pr^2^.

**Figure 2:**
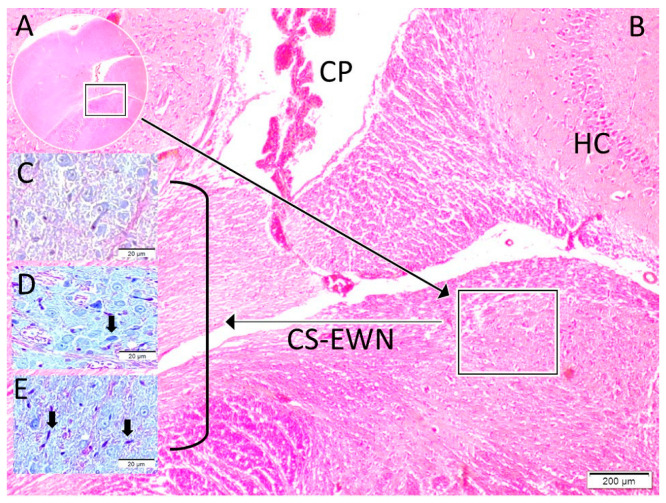
Histological Localization and Degeneration in Edinger–Westphal Nucleus. (**A**,**B**): Placement of superior colliculus in mesencephalic tegmentum adjacent to hippocampus (HC, boxed). (**B**): Magnified localization of Edinger–Westphal nucleus (CS-EWN) in superior colliculus (CS), neighbouring ventricles, choroid plexuses (CP), and hippocampus (HC). (**C**–**E**): Normal (Control/(**C**)), mild (SHAM/(**D**)), and advanced degenerated neurons (black arrows, Study/(**E**)) in Edinger–Westphal nucleus. Criteria for neuronal degeneration: cellular angulation, nuclear shrinkage, cytoplasmic condensation, and darkening (LM, H&E; ×10 for (**D**), ×40 for (**A**–**C**); scale bars as indicated).

**Figure 3:**
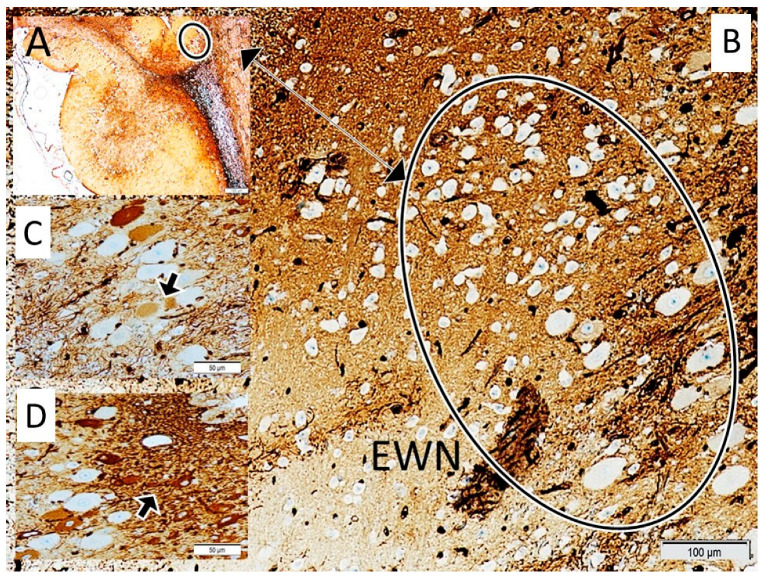
Anatomical Localization of the Superior Colliculus and Edinger–Westphal Nucleus with Neuronal Degeneration Analysis. The image shows the anatomical location of the superior colliculus (within the circle/(**A**)) in the mesencephalic tegmentum of a normal specimen (**A**). The localization of the Edinger–Westphal nucleus (EWN) within the superior colliculus is also depicted. Normal (Control/(**B**)), mildly degenerated (SHAM/(**C**)), and severely degenerated neurons (indicated by black arrows) are observed in the Edinger–Westphal nucleus across normal (**B**), sham (**C**), and study group subjects (**D**). Criteria for neuronal degeneration generally include cellular/neuronal angulation, nuclear shrinkage, cytoplasmic condensation, and cellular darkening (LM, GFAP, ×10/(**D**) ×40/(**C**,**D**)).

The normal and degenerated neuron densities within the Edinger–Westphal nucleus were subsequently quantified using stereological methods. Furthermore, degenerated neuron densities of the Edinger–Westphal nucleus (n/mm^3^), pupil diameters (micrometres), and pupil index values (PIV) were compared between the groups (Figure 1, Figure 2 and Figure 3). The pupil index value, referred to as the DADA index, was defined as the ratio of the iris surface area to the pupil surface area and calculated using the formula: PIV(DADA-I) = (IRr^2^ − Pr^2^)/Pr^2^ (where IR represents the radius of the iris and Pr represents the radius of the pupil). This formula is visually represented in Figure 1.

Statistical analysis involved the comparison of the three independent groups (control, sham, and study groups) for three key parameters/variables: degenerated neuron densities of the Edinger–Westphal nucleus, pupil diameters, and pupil index values. Group comparisons were performed using the Kruskal–Wallis test, an appropriate non-parametric test for comparing more than two independent samples. In cases where the Kruskal–Wallis test indicated a statistically significant difference, Bonferoni-corrected Mann–Whitney U tests were conducted as post hoc analyses to identify specific group differences. Additionally, Spearman correlation analysis was performed to investigate relationships between these variables.

## 3. Results

This study assessed pupil diameter, Dangerous Alarming Diameter Assessment (DADA) index, and degenerated neuron density in the Edinger–Westphal nucleus (EWN) across three groups: Control (*n* = 5), Sham (*n* = 5), and Study (subarachnoid haemorrhage, SAH, *n* = 12). Descriptive statistics, pairwise comparisons, and correlation analyses are presented in Table 1 and Table 2

For pupil diameter, mean values were 5900.00 ± 546.32 µm in the Control group, 6310.00 ± 533.35 µm in the Sham group, and 9989.50 ± 747.12 µm in the Study group. Median (min–max) values were 5900.00 (5200.00–6600.00) µm, 6340.00 (5452.00–6850.00) µm, and 10,039.00 (8780.00–11,138.00) µm, respectively. Pairwise comparisons revealed no significant difference between Control and Sham groups (*p* = 0.602), but significant differences were observed between Control and Study (*p* < 0.001) and Sham and Study (*p* < 0.001) groups, indicating a marked increase in pupil diameter following SAH.

For the DADA index, mean values were 2.32 ± 0.43 in the Control group, 1.95 ± 0.37 in the Sham group, and 1.06 ± 0.31 in the Study group. Median (min–max) values were 2.32 (1.78–2.90), 1.95 (1.47–2.41), and 1.04 (0.58–1.65), respectively. Significant differences were found between Control and Study (*p* = 0.003) and Sham and Study (*p* = 0.008) groups, but not between Control and Sham (*p* = 0.444), suggesting that the DADA index is sensitive to SAH-induced changes.

For degenerated neuron density in the EWN, mean values were 3.00 ± 0.95 n/mm^3^ in the Control group, 12.00 ± 2.92 n/mm^3^ in the Sham group, and 18.00 ± 4.00 n/mm^3^ in the Study group. Median (min–max) values were 3.00 (1.80–4.20), 12.00 (8.20–15.80), and 18.00 (11.00–25.00), respectively. Significant differences were observed between Control and Sham (*p* = 0.004), Control and Study (*p* < 0.001), and Sham and Study (*p* = 0.018) groups, reflecting progressive neurodegeneration post-SAH.

Correlation analyses demonstrated strong negative correlations between pupil diameter and degenerated neuron density (r = −0.972, *p* < 0.001, *N* = 12) and between DADA index and degenerated neuron density (r = −0.977, *p* < 0.001, *N* = 12). These findings indicate that increased pupil diameter and decreased DADA index values are strongly associated with higher EWN neurodegeneration. Notably, pupil diameter assessment classified all 12 subjects in the Study group as deceased, primarily due to fixed and dilated pupils. However, evaluation using the DADA index identified only 8 of these 12 subjects as deceased, with EWN degenerated neuron density exceeding 80% in these cases. In the remaining 4 subjects, the DADA index revealed EWN degenerated neuron density below 80%, indicating that these subjects were not deceased despite being classified as such by pupil diameter assessment. This underscores the DADA index’s superior precision in distinguishing viable subjects from those with severe neurodegeneration, offering a more refined diagnostic tool for brain death assessment. The DADA index’s ability to accurately reflect EWN neurodegeneration below the 80% threshold highlights its potential as a robust criterion for brain death, with implications for future diagnostic advancements, including brain transplantation.

## 4. Discussion

The results of this experimental investigation give strong support that the Dangerous Alarming Diameter Assessment (DADA Index) has better dependability than traditional pupil diameter measures in evaluating neurological impairment following subarachnoid haemorrhage. With statistical significance that greatly surpassed the correlations seen with pupil diameter measurements alone, our findings showed a notable inverse relationship between the DADA Index values and the density of degenerated neurones in the Edinger–Westphal nucleus (EWN). This strong link implies that the DADA Index is a more sensitive indicator of parasympathetic neural circuit integrity than traditional measures [31,32]. Building on this evidence, the remarkable drop in DADA Index values seen in the experimental SAH group (1.023 ± 0.324) relative to control (2.32 ± 0.421) and SHAM (1.946 ± 0.376) groups shows the severe neurological effect of subarachnoid haemorrhage on the pupillary control systems. This result is consistent with earlier findings [33,34], who found that clinical outcomes following aneurysmal subarachnoid haemorrhage were highly related to pupillary anomalies. Our research, on the other hand, broadens this knowledge by offering a measurable criterion that may be standardised across various patients and clinical environments, hence perhaps solving the long-standing problem of interobserver variation in pupillary evaluations [35,36]. Further enhancing our understanding, essential to the reading of these findings is the realisation that the DADA Index basically measures the correlation between iris surface area and pupil surface area, which naturally reflects the functional balance between sympathetic and parasympathetic neurological control systems [9,37]. Although traditional pupillary evaluation emphasises just the absolute diameter of the pupil, this traditional method overlooks individual differences in iris anatomy and the relative proportion of pupillary to ciliary zones, which directly affects the seen pupillary reaction [38].

The structural organisation of the iris itself provides the anatomical foundation for the enhanced predictive value of the DADA Index. The iris consists of separate functional zones, approximately the pupillary zone (making up about 25% of iris surface area) and the ciliary zone (comprising around 75%), as shown in thorough iris anatomy research by Naumann and further explained by Ortube and colleagues [39,40]. These zones not only vary in structural makeup but also show various neural control mechanisms, the pupillary zone under parasympathetic control via the EWN and the ciliary zone controlled by sympathetic innervation via the superior cervical ganglion [9,41]. Building on this anatomical framework, the DADA Index is therefore critically physiologically based on this anatomical divide. Neurological damage, especially affecting the EWN following subarachnoid haemorrhage, results in a loss of parasympathetic control that produces a measurable change in the pupillary to ciliary zone ratio. This change is recorded by the DADA Index but not sufficiently shown in simple pupil diameter measurements [42,43]. Loewenfeld and Lowenstein’s (1993) [44,45] study, performed decades ago, showed that various neurological diseases may generate same pupil diameters by means of various pathophysiological processes, hence compromising the reliability of solely diameter-based evaluations. Further deepening this insight, our experimental results show that the DADA Index falls in a consistent and measurable way as EWN neuronal degeneration following subarachnoid haemorrhage rises. This is an indirectly but very consistent indicator of the sympathetic-parasympathetic balance controlling pupillary function [46]. Bremner and Maddess [47,48] study on pupillary physiology backs up our finding that relative changes in pupillary vs. ciliary zone proportions provide more clinically meaningful data than absolute pupil size by itself. Extending this analysis, this negative association seen between EWN neuronal degeneration and DADA Index values across all experimental individuals implies that this measure represents a fundamental neurophysiological link rather than merely an epiphenomenon. Kozicz and Park’s [49,50] neuroanatomical research has revealed the EWN’s susceptibility to many types of damage, including ischaemia, compression, and inflammatory processes linked to subarachnoid haemorrhage. Our results imply that the DADA Index offers a window into these pathophysiological processes not accessible by traditional pupillary evaluation.

Traditional pupillometry does not measure the balance between sympathetic and parasympathetic modulation of pupillary function, but the DADA Index does. It is more sophisticated because it examines the relationship between the iris’s pupillary and ciliary zones to capture this balance [51]. Building on this conceptual advance, McDougal and Gamlin [51,52] have well-established the importance of sympathetic-parasympathetic balance in neurological evaluation by showing that different neurological diseases generate unique patterns of autonomic disturbance detectable by advanced pupillary examination. Our results add to this knowledge by indicating that the DADA Index substantially corresponds with histological alterations in the EWN, hence implying that it correctly indicates parasympathetic system integrity after neurological damage. Further supporting this evidence, studies on the autonomic regulation of pupillary function by Peinkhofer and et al. [46] back up our finding that ratio-based measures offer better information on underlying neurological function than basic diameter measurements. Their studies showed that different clinical conditions cause certain changes in the connection between iris and pupil sizes that mirror certain autonomic dysfunction patterns. The DADA Index uses this correlation to offer a consistent, measurable tool that reflects these small but clinically relevant variations. Extending this physiological insight, the multiple neuronal routes regulating various facets of pupillary function underlies the physiological foundation of this link. Sympathetic stimulation mostly affects the dilator muscles of the ciliary zone, while parasympathetic stimulation operates more on the sphincter muscles of the pupillary zone, as noted by Szabadi [53] in a thorough study of pupillary control mechanisms. By measuring the ratio between these functionally different zones, the DADA Index offers a physiologically based indirect evaluation of their relative activity, so providing insights into the underlying neurological integrity not accessible by conventional pupillary assessment [54].

**Figure 4:**
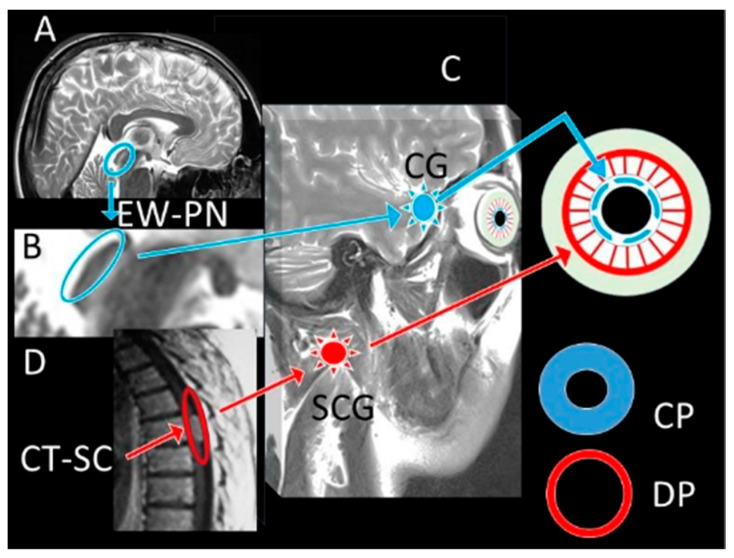
Table of Pathogenesis: Pathogenesis Table: Human brain MRI; the location of the Edinger Westphal and Perlia nuclei (EW-P) located in the tegmentum mesencephali in the superior colliculus (**A**,**B**), their connection with the ciliary ganglion network (CG) and pupillary muscles (Blue arrows), the location of the parasympathetic pupilloconstrictor fibres and muscles affecting the pupilloconstrictor muscles that innervate the pupillae (Blue/(**A**,**B**)) (**C**); the postganglionic fibres going to the pupillodilator muscles (Red arrow/(**B**)) of the sympathetic cervical ganglion network (SCG) originating from the cervicothoracic sympathetic centre (CT-SC) and coming to the superior cervical sympathetic ganglion (SCG), and the pupillodilator muscles they affect are observed (**D**). While the pupils contract with the parasympathetic effect (CP), they dilate with the sympathetic effect (DP). Representative constricted pupil (Blue Ring) and dilated pupil (Red Ring) are seen.

These results have far-reaching clinical consequences. In critical care environments, current neurological assessment techniques depend mostly on qualitative or semi-quantitative pupil assessments vulnerable to significant interobserver variation [55,56]. A standardised, ratio-based measure like the DADA Index will greatly increase the correctness and consistency of pupillary evaluation across many different clinical environments. İn the studies conducted [16,25,57] found that even small increases in the accuracy of neurological evaluation could lead to notable changes in clinical results by means of early intervention and more suitable treatment techniques. Building on this potential, the DADA Index has possible benefits in both initial assessment and continuous monitoring in the particular setting of subarachnoid haemorrhage, when fast and precise neurological assessment is critical. Early identification of neurological degeneration following SAH has been shown to greatly enhance results, especially when intervention takes place before permanent damage. Unlike traditional pupillary evaluation, the DADA Index could offer a more sensitive early warning system that might enable earlier identification of rising intracranial pressure, imminent herniation, or progressive brainstem compression. Moreover, the DADA Index could be used for other acute neurological disorders outside SAH. Boulter and et al. [58] research on pupillary alterations in traumatic brain injury indicates that more complex pupillary measurements may increase predictive accuracy. Feng and et al. [59] likewise showed that in post-cardiac arrest patients with hypoxic–ischemic encephalopathy, quantitative pupillary evaluation enhances outcome prediction. By offering a more physiologically based measure that considers individual differences in eye anatomy, the DADA Index might improve these evaluations. Further extending its implications, our results imply, perhaps most importantly, that the DADA Index could be a useful complement to present brain death criteria. Though present techniques lack consistency and are influenced by other variables, pupillary evaluation stays a foundation of the clinical examination, as Wijdicks and et al. [60] point out in their thorough study of brain death diagnosis. By measuring the correlation between iris and pupil surface areas, the DADA Index could offer a more consistent and objective sign of total parasympathetic pathway failure, hence lowering doubt in these vital decisions. Furthermore, beyond the identification of parasympathetic failure, the potential of the DADA Index extends to its critical role in the differential diagnosis of pathologies involving isolated or combined sympathetic and parasympathetic system damage. The DADA-I’s primary strength lies in this differential diagnosis. Circulatory disturbances affecting the oculomotor complex and EWN (e.g., midbrain ischemia) lead to parasympathetic failure and pupillary dilation, causing the DADA-I value to decrease. Conversely, pathologies of the cervicothoracic spinal cord, which lead to sympathetic failure, result in unopposed parasympathetic dominance and excessive miosis, causing the DADA-I value to increase. While the literature focuses on ‘brain death,’ it often neglects ‘spinal cord death’ and the concept of ‘cerebrospinal death.’ Whereas pupil diameter alone can be misleading, DADA-I accounts for both the iris (sympathetic) and pupil (parasympathetic) areas. It thus offers a more reliable tool for understanding the pathology in craniospinal trauma patients: a decrease in DADA-I suggests cranial pathology, while an increase suggests spinal pathology (Figure 4). This is in line with Lenga and colleagues’ [61,62] recent work advocating for more exact physiological measures in brain death diagnosis. Building on this standardisation potential, in addition to these insights, this study’s findings suggest that the DADA Index could serve as a valuable tool in standardising pupillary assessments across different practitioners and institutions.

Building on these findings, this work offers the realm of neurocritical care and neurological evaluation numerous notable additions. First, it presents and validates a new measure, the DADA Index, that addresses historical constraints in conventional pupillary assessment by considering individual differences in ocular anatomy and emphasising the physiologically relevant ratio between iris and pupil surface areas instead of absolute diameter measurements [63]. Second, this study offers insightful analysis of the pathophysiological mechanisms underlying pupillary abnormalities following subarachnoid haemorrhage by directly linking a clinically observable measure, the DADA Index, to histopathological changes in neuronal degeneration in the EWN [36]. Third, the work adds to the developing area of quantitative pupillometry by proving that complex analysis of pupillary measures can produce clinically pertinent information beyond that accessible by current evaluation techniques [55,64].

Transitioning to future directions, although this experimental investigation offers strong proof of the DADA Index’s use in evaluating neurological impairment following subarachnoid haemorrhage, several significant avenues for further study become clear. Establishing the applicability and dependability of the DADA Index in human participants across various clinical environments requires first clinical validation studies. To create normative data and measure individual variability, these studies should comprise baseline examinations in healthy populations as well as acute neurological diseases. Building on this foundation, longitudinal research looking at the predictive efficacy of the DADA Index in forecasting results after subarachnoid haemorrhage would first offer insightful analysis of its therapeutic relevance. Bower and colleagues’ [55] work has shown the prognostic utility of quantitative pupillometry in neurocritical care; analogous methods should be used to assess if the DADA Index provides better prognostic information than current measures. İt is absolutely vital that technology evolve to enable quick, dependable calculation of the DADA Index in clinical environments. Specialised software and hardware solutions that can precisely measure both pupil and iris sizes and automatically compute the DADA Index would help clinical implementation and standardisation by building on developments in automated pupillometry described by Couret and colleagues [3]. İn this context; our study on the possible use of the DADA Index as an additional factor for brain death decision merits focused effort. Wijdicks and colleagues [60] point out that clinical neurology still has a major aim in brain death determination: better objectivity and dependability; the DADA Index could provide benefits that should be methodically investigated in relevant clinical environments. Extending this scope, also more research on the DADA Index’s association with other neurological diseases beyond subarachnoid haemorrhage would assist to define its generalisability and possible uses throughout the whole range of neurocritical care. The work of Peinkhofer et al. [46] implies that various neurological diseases create unique patterns of pupillary abnormalities; investigating whether the DADA Index improves identification and distinction of these patterns is a significant avenue for future study.

While this study provides valuable insights, it is important to acknowledge its limitations. The experimental design utilised a rabbit model, which may not fully replicate the complex physiological responses observed in human subjects, potentially limiting the generalizability of the findings to clinical practice. Additionally, the sample size was relatively small, consisting of only a limited number of animals across the experimental, control, and sham groups, which could affect the statistical power and reliability of the results.

## Figures and Tables

**Table 1:** Descriptive Statistics and Pairwise Comparisons Between Groups.

		Control (1)	SHAM (2)	STUDY (3)	*p* (1–2)	*p* (1–3)	*p* (2–3)
Pupil Diameter	Mean ± SS	5900.00 ± 546.32	6310.00 ± 533.35	9989.50 ± 747.12			
	Median (Min–Max)	5900.00 (5200.00–6600.00)	6340.00 (5452.00–6850.00)	10,039.00 (8780.00–11,138.00)	0.602	<0.001 *	<0.001 *
DADA Index	Mean ± SS	2.32 ± 0.43	1.95 ± 0.37	1.06 ± 0.31			
	Median (Min–Max)	2.32 (1.78–2.90)	1.95 (1.47–2.41)	1.04 (0.58–1.65)	0.444	0.003 *	0.008 *
Degeneration Neuron	Mean ± SS	3.00 ± 0.95	12.00 ± 2.92	18.00 ± 4.00			
	Median (Min–Max)	3.00 (1.80–4.20)	12.00 (8.20–15.80)	18.00 (11.00–25.00)	0.004 *	<0.001 *	0.018 *

*: Significant level < 0.05; *p*: Bonferoni-corrected Mann–Whitney U tests.

**Table 2:** Correlation Analysis Between Variables in the Study Group.

		Pupil Diameter	Degeneration Neuron
Dada Index	r	−0.972 **	−0.977 **
	*p*	<0.001	<0.001
	*N*	12	12

r: Spearman Correlation Coefficient, *p*: Significant (2-tailed); *N*: count. ** Correlation is significant at the 0.01 level (2-tailed).

## Data Availability

The original contributions presented in the study are included in the article, further inquiries can be directed to the corresponding author.
